# On site DNA barcoding by nanopore sequencing

**DOI:** 10.1371/journal.pone.0184741

**Published:** 2017-10-04

**Authors:** Michele Menegon, Chiara Cantaloni, Ana Rodriguez-Prieto, Cesare Centomo, Ahmed Abdelfattah, Marzia Rossato, Massimo Bernardi, Luciano Xumerle, Simon Loader, Massimo Delledonne

**Affiliations:** 1 Tropical Biodiversity section, Science Museum of Trento, Trento, Italy; 2 Personal Genomics s.r.l., Verona, Italy; 3 Functional Genomics Center, Department of Biotechnology, University of Verona, Verona, Italy; 4 Biogeography Research group, Department of Environmental Sciences, University of Basel, Basel, Switzerland; 5 Department of Life Sciences, University of Roehampton, London, United Kingdom; Midwestern University, UNITED STATES

## Abstract

Biodiversity research is becoming increasingly dependent on genomics, which allows the unprecedented digitization and understanding of the planet’s biological heritage. The use of genetic markers *i*.*e*. DNA barcoding, has proved to be a powerful tool in species identification. However, full exploitation of this approach is hampered by the high sequencing costs and the absence of equipped facilities in biodiversity-rich countries. In the present work, we developed a portable sequencing laboratory based on the portable DNA sequencer from Oxford Nanopore Technologies, the MinION. Complementary laboratory equipment and reagents were selected to be used in remote and tough environmental conditions. The performance of the MinION sequencer and the portable laboratory was tested for DNA barcoding in a mimicking tropical environment, as well as in a remote rainforest of Tanzania lacking electricity. Despite the relatively high sequencing error-rate of the MinION, the development of a suitable pipeline for data analysis allowed the accurate identification of different species of vertebrates including amphibians, reptiles and mammals. *In situ* sequencing of a wild frog allowed us to rapidly identify the species captured, thus confirming that effective DNA barcoding in the field is possible. These results open new perspectives for real-time-on-site DNA sequencing thus potentially increasing opportunities for the understanding of biodiversity in areas lacking conventional laboratory facilities.

## Introduction

The scientific community is in agreement that we are in the midst of the sixth great mass extinction [1]. This has been attributed to the modification and destruction of natural habitats by humans, placing a wide range of organisms at risk [1–3]. Although the loss of biodiversity is global, the geographic patterns of species loss are non-random [4]. The number of species in decline per 10,000 km^2^ (IUCN population status ‘decreasing’) varies regionally, with the highest numbers in tropical areas even after factoring in the greater species diversity [4]. Many species in tropical countries are declining to the point of extinction. To mitigate these losses requires, among other actions, the rigorous evaluation of biodiversity and an appropriate resource allocation during conservation planning. The latter is typically based on the evaluation of species numbers in a given area, reflecting taxonomic richness and endemism [5]. This can lead to the designation of protected areas or the identification of areas with biological value and thus deserving specific conservation efforts [6]. More recently, conservation efforts have focused also on the preservation of the underlying functional and genetic diversity that the different species represent [7].

Despite the significant progress in theoretical and applied conservation science, the assessment of conservation priorities is hampered by the knowledge gap on biodiversity. This has been described as the Darwinian shortfall [8], *i*.*e*. phylogenies for most groups of organisms remain unresolved, and the Linnean and Wallacean shortfalls, *i*.*e*. no-one knows how many species inhabit our planet and how they are distributed [9]. This knowledge gap implies that our understanding of species diversity is incomplete, and due to the time-consuming traditional approaches applied by taxonomists, the complexity and diversity of nature will not be fully appreciated in the short time remaining before the extinction of many species. Traditionally, species identification relied on the morphological characterization of the organism [10]. However, with the advances in molecular techniques in the last decades, the use of genetic markers, also known as DNA barcodes, now represents the state-of-art approach for the identification of novel species [11]. The term “DNA barcoding” was first mentioned in 2003 by Hebert et al. (2003), that proposed the use of the mitochondrial gene, cytochrome *c* oxidase I (*CO1*), as an identification system for animals [11]. DNA barcoding provides an effective tool to investigate biodiversity [12] due to its rapid, efficient, and cost effective features, that allows an objective identification of species by expert and non-expert taxonomists alike [13]. For these reasons, DNA barcoding has been intensively utilized in many research fields, and subsequently other barcodes were developed to identify, not only animals, but also other organisms [14, 15]. One of the most popular example of alternative barcodes is the 16S gene, encoding a subunit of the rRNA. This barcode, in addition to being very much used for prokaryotes i.e. archaea and bacteria [16], it was proved to be superior to COI in some major vertebrate clades [17] For years, Sanger sequencing was the method used to sequence DNA barcodes, mainly because it was the only available platform, but also for its high accuracy. With the advent of the massive parallel sequencing *e*.*g*. solid-state-, pyro-, and semiconductor-sequencing methods, several platforms became available and represent valid alternatives to the Sanger method. Despite the sequencing platform used, a major drawback of barcoding is the need for a dedicated laboratory, whereas many important sites for biological conservation are remote and inaccessible [18]. Furthermore, the legal procedures governing the transport of biological material between biodiversity-rich and resource-rich nations vary from country to country, making difficult to transport samples of native species outside the country of collection. Strategies to increase the amount of genetic and genomic data produced in biodiversity-rich countries are therefore a high priority–as already demonstrated by the portable genome sequencing for Ebola surveillance [19].

As participants in the MinION early Access Program (MAP), we tested the Nanopore DNA sequencing platform developed by Oxford Nanopore Technologies (ONT) (Oxford, UK), and contributed to its last-phase development before the launch into the general market. Being a technology under development, the nanopore-based sequencing still suffer some drawbacks, as for example a high error rate [20]. Despite these limits, the MinION platform offers big potential advantages in the context of biodiversity research, *i*.*e*. portability and low costs of instrument and reagents. Unlike other sequencing technologies, the MinION is therefore not restricted to laboratories and can be used by research groups located far from the nearest sequencing facilities [19].

In this context, the aim of the present work was to construct and validate a portable laboratory based on the MinION platform to be used for the sequencing in the field. The study investigated the capability of the MinION to produce accurate sequences of standard barcodes utilized for the identification of vertebrates and assessed an appropriate data analysis pipeline to exploit the MinION data for species barcoding. End result was the set-up of a miniaturized portable kit validated under extreme tropical environmental conditions that can be used to identify previously known and un-known vertebrate species and, potentially, to address the biodiversity knowledge gap.

## Materials and methods

### Ethics statement

The work conducted as well as the sampling procedures utilized in the field were approved the Tanzania Commission for Science and Technology and the Tanzania Wildlife Research Institute (TAWIRI) under the permit N° RCA 2014–338. The field studies did not involve endangered or protected species. The individual in this manuscript has given written informed consent (as outlined in PLOS consent form) to publish these case details.

### Set-up of the portable laboratory

To conduct a sequencing experiment in the field, most of lab equipment and reagents were optimized, and/or replaced, with portable, user friendly and stable reagents that could perform properly in the field. The selection of suitable devices, reagents and protocols was conducted in collaboration with (Biodiversa S.R.L., Trento, Italy). For the amplification and quantification of DNA, we used the GeneOne device (Biodiversa S.R.L., Trento, Italy), which consists of a thermocycler and a fluorometer with two excitation wavelengths (490 and 535 nm) and two emission filters (520 and 560 nm). In addition, to avoid the need for cumbersome and energy demanding equipment such as centrifuges, large refrigerators/freezers, and electrophoresis apparatus, all devices requiring electricity were modified to receive power from 12V portable batteries. The portable MinION device developed by Oxford Nanopore Technologies (ONT) (Oxford, UK) was selected as sequencing machine given its small dimensions, *i*.*e*. 10 x 2.2 x 3.2 cm, and the minimal requirement of power supply (USB connection). Furthermore, protocols and reagents were selected to avoid the need for storage at -20°C. All PCR reagents were lyophilized (Sentinel S.R.L., Milan, Italy) and stable at room temperature, while sequencing reagents were conserved in a portable 4°C refrigerator. Finally, since the library preparation protocol, recommended by ONT, consists of end-repair and dA-tailing steps, which require enzymes that need storage at -20°C, we tested 3 different protocols with the aim to avoid the need of these reagents, as well as reduce the library preparation time. Protocol 1 was used as recommended by ONT *i*.*e*. including end-repair and dA-tailing steps. Protocol 2 omits these steps, and protocol 3 was exactly like protocol 2 except that phosphorylated primers were used in the barcode amplification.

### DNA extraction and amplification

Five distinct organisms, kindly provided by the Trento Science Museum (MUSE), were used for the validation of the portable sequencing laboratory and the analysis of barcoding results (Table 1). The sixth organism analyzed (*Arthroleptis xenodactyloides*) was collected and analyzed in a montane rainforest of central-south Tanzania.

**Table 1:** Summary of the species studied in the present work, their origin, tissue sampled, and the gene analyzed.

Species	Origin	Tissue	Gene analyzed
*Amietophrynus brauni*	Tanzania-MUSE	phalanx	16S
*Leptopelis vermiculatus*	Tanzania-MUSE	phalanx	16S, CO1
*Rieppeleon brachyurus*	Tanzania-MUSE	connective skin tissue	16S
*Sorex alpinus*	Italy-MUSE	connective skin tissue	16S
*Rhynchocyon udzungwensis*	Tanzania-MUSE	connective skin tissue	CO1
*Arthroleptis xenodactyloides*	Tanzania	blood	16S

Total DNA was extracted from a 2-mm tissue fragment or 2 μl of blood treated with 100ul DNAzol at 80°C for 15 minutes (Molecular Research Center, Cincinnati, USA). 1 μl of debris-free DNAzol homogenate was directly used in PCR amplification reactions without any further processing. PCR amplification of the barcodes was carried out in 25 μl reactions comprising 400 nM of each primer, 2 mM MgCl_2_ and 1 unit *Taq* Polymerase and buffer components (Sentinel S.R.L., Milan, Italy) previously re-suspended in 24 μl milliQ water according to the manufacturer’s instructions. PCR reactions were conducted in the GeneOne portable PCR device with the 5’-end phosphorylated primer pairs reported in Table 2. For protocol 1 and 2, the 16S gene was also amplified using regular, non-5’-end phosphorylated primers. The 16S genes of all amphibians analyzed in the study were amplified with 16SAR forward and reverse primers, using the following thermocycler program: 95°C for 3 min followed by 33 cycles of 95°C for 20 s, 52°C for 20 s and 72°C for 30 s, with a final 3 min extension at 72°C [21, 22]. The mitochondrial gene CO1 of the frog *Leptopelis vermiculatus* was amplified using forward and reverse primer Amp-P3 F and Amp-P3 R, respectively. The amplification cycle consisted of a cycle at 95°C for 3 min followed by 35 cycles of 95°C for 40 s, 45°C for 30 s and 72°C for 40 s, with a final 5 min extension at 72°C [23]. Finally, the CO1 of the giant sengis, *Rhynchocyon udzungwensis* was amplified with the LCO1490 and HC02198: 94°C for 1 min followed by 5 cycles of 94°C for 1 min, 45°C for 1 min and 72°C for 1 min, followed by 35 cycles of 94°C for 1 min, 50°C for 1 min and 72°C for 1 min, with a final 5 min extension at 72°C [24]. PCR products were purified using Agencourt AMPure XP beads at 1.8: 1 beads to DNA ratio (Beckman Coulter Inc. Pasadena, USA). The PCR products were quantified using the fluorometer integrated in the GeneOne device.

**Table 2:** Primer pairs used for the amplification of the selected barcode genes.

Species	Primer name	Forward 5'-3'	Gene	Amplicon length	Reference
vertebrates	16Sar-5'	CGCCTGTTTATCAAAAACAT	16S	~600bp	[21, 22]
	16S	CCGGTTTGAACTCAGATCA			
*Leptopelis vermiculatus*	Amp-P3 F	CAATACCAAACCCCCTTRTTYGTWTGATC	CO1	~900bp	[23]
	Amp-P3 R	GCTTCTCARATAATAAATATYAT			
*Rhynchocyon udzungwensis*	LCO1490	GGTCAACAAATCATAAAGATATTGG	CO1	~710bp	[24]
	HC02198	TAAACTTCAGGGTGACCAAAAAATCA			

### Library preparation and MinION sequencing

DNA libraries were prepared from 1.5μg of the purified PCR products. Double strand DNA molecules were end-repaired using the NEBNext End Repair Module (New England Biolabs, Ipswich, USA), followed by purification with Agencourt AMPure XP at 1.8:1 beads to DNA ratio. Only in the case of protocol 1, the purified amplicons were then processed using the NEBNext dA-tailing module (New England Biolabs), these steps were skipped in protocol 2 and 3. Amplicons from the three protocols were used to prepare sequencing libraries using the ONT DNA Sequencing kits (SQK-MAP004, SQK-MAP005, and SQK-MAP006). 2 μl of sequencing Hairpin HP Adapter (HPA) were ligated using 50 μl Blunt/TA Ligase Master Mix (New England Biolabs, Ipswich, USA) in the presence of 10 μl Adapter Mix and incubated for 10 minutes at room temperature. The adapter mix HPA consists of a linear double strand sequence and a hairpin sequence that links the positive and negative strand of each fragment to allow the sequencing of both strands (2D reads). After adapter ligation, the library was conjugated with 1μl of Hairpin Tether (HPT) motor protein to allow the passage of the fragment through the nanopore on the flowcell. After 10 minutes at room temperature, the ligated DNA was cleaned up using 1X Dynabeads^®^ MyOne^™^ Streptavidin C1 (Thermo Fisher Scientific, Waltham, USA) that can select the library by binding the biotins conjugated to the adapters. Prior to sequencing, a quality control was carried out on the MinION flowcell to determine the number of available pores for the sequencing. Before library loading, the flow cell was primed using 500 μL of Priming Mix (500 μL running buffer (RNB) buffer, 473.4 μL nuclease-free water and 26.46 μL fuel mix (FMX) buffer) twice with 10 minutes of incubation after each addition. The sequencing library mix, was prepared by combining 8μL of library, corresponding to about 200ng, with 75μL of RNB buffer, 5.3 μL of FMX, and 65 μL nuclease-free water. The sequencing analysis from *A*.*brauni* and *A*. *xenodactyloides* were obtained using the MAP-005 kit, while the other samples were analyzed with the MAP-006 kit reflecting an update provided by the manufacturer. The sequencing run was performed for 6 to 16 hours using the “MAP_48Hr_Sequencing_Run_SQK_MAP00X” protocol using the MinKNOW software. To test the new MinION chemistry, libraries were prepared using the SQK-LSK208 kit and flowcell were run with the program “NC_48Hr_Sequencing_Run_FLO_MIN106_SQK-LSK208”. Raw MinION reads produced in the study are reported in S2 File.

### Sanger sequencing

Amplicons sequenced using the MinION platform were analyzed in parallel with the Sanger method to confirm the identity of the tested organism and evaluate the performance of the new system. DNA sequencing was performed with fluorescently labeled dideoxy terminators on an ABI 3730XL Genetic Analyzer (Applied Biosystems) at the facilities of BMR Genomics (Padova, Italy). Sequences were visualized and edited using Chromas version 1.45 [25]. Raw Sanger data produced in the study are reported in S2 File.

### Data analysis

Bases were called from raw MinION data using the Metrichor Agent with the 2D Basecalling workflow. The fasta files were extracted using poretools [26]. For downstream analysis, we adapted a previously described pipeline that relies on using *de novo* assembly of MinION reads [27]. Loman’s method uses the overlapping regions which are detected between reads using DALIGNER [28], which are then corrected by a multiple-alignment process using Partial Order Aligner [29]. The corrected reads are assembled using the Celera Assembler [30], producing a *de novo* assembled consensus of the entire dataset. Scripts used for the reconstruction of a consensus sequence from MinION sequencing reads are reported in S3 File.

**Fig 1:**
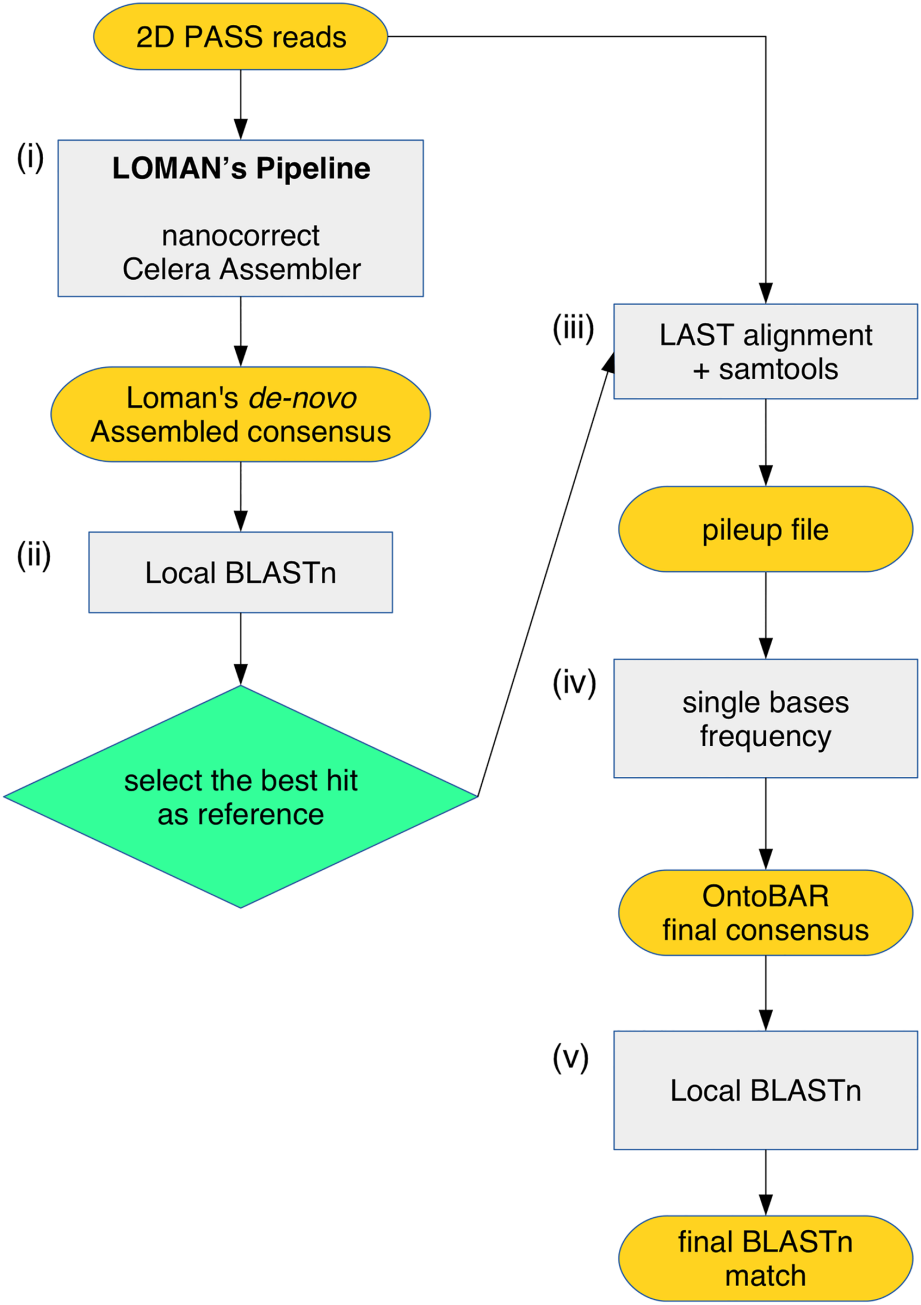
The “ONtoBAR” pipeline. (i) The 2D Pass reads produced with the MinION are assembled de-novo using the Loman’s method; (ii) the obtained Loman’s consensus sequence is then BLASTed to retrieve the most similar sequence present in the NCBI database; (iii) the best hit is then selected as the new reference to which the initial 2D Pass reads are aligned using LAST; (iv) the frequency of each nucleotide is calculated for every position along the reference sequence and the final ONtoBAR consensus is generated and (v) BLASTed vs the NCBI database.

The error-rate of the reads produced by the MinION could be higher than the acceptable interspecies variation, which could make the identification of the investigated organism at the species level more difficult. In addition, alignment software are designed to maximize alignments by adapting sequence reads to the reference. Given these issues, we therefore tested whether the correct consensus sequences could be called even when the reference differed slightly from the DNA sequence of the species under investigation. At this aim additional steps to Loman’s method were added, in which the final assembly of the barcode gene is BLASTed locally against the NCBI nucleotide (nt) database using BLAST [31], and the most similar sequence *i*.*e*. the best BLAST hit, was (i) retrieved from the NCBI nt database and (ii) used as a reference sequence to reconstruct a final consensus from the initial set of raw MinION reads using LAST. Starting from the binary sequence alignment data, a pileup file was created using SAMTOOLS [32], and the frequency of each nucleotide per reference position was calculated using a custom-made Python script (S4 File) that parsed the pileup file. Finally, the final consensus is then BLASTed again against NCBI nt database using BLAST software. This method is designated here as “ONtoBAR pipeline” (Fig 1).

## Results

### Validation of the portable laboratory

In order to identify the most suitable protocols for a portable sequencing laboratory to be used in the context of a tropical forest, we evaluated: 1) the effect of storage temperature on the MinION reagents and flowcells, 2) the impact of tropical forest conditions on the sequencing performance 3) the impact of the protocol shortening on the sequencing performance 4) the suitability of MinION for DNA barcoding. The amplicon of the 16S gene of the toad *Amietophrynus brauni* was used as starting material for the validation experiments.

#### Storage temperature

To verify the impact of storage temperature on the sequencing kit, we compared the sequencing output and quality after storing the ONT DNA Genomic kit at 4°C for one week, instead of -20°C as recommended by the company (Table 3). The sub-optimal storage of reagents slightly reduced the number of total and 2D normalized reads obtained (reduction of 78 and 18 reads on average, respectively). Still, the storage at 4°C did not affect the quality of sequencing given that the percentage of 2D reads generated did not vary significantly between the two conditions tested (Table 3).

**Table 3:** Impact of storage temperature on sequencing performances.

			*Raw Reads*			*Normalized Reads*		
*Storage Temp*	Channels QC	Channels with Reads	Total	2D	Pass 2D	Total	2D	Pass 2D
*-20°C*	262	226	54380	7873 (14.4%)	2163 (4.0%)	241	35	10
*-20°C*	494	425	141908	33200 (23.4%)	8144 (5.7%)	334	78	19
*+4°C*	120	128	11594	2307 (19.9%)	784 (6.8%)	91	18	6
*+4°C*	365	353	115673	20529 (17.7%)	5652 (4.9%)	328	58	16

Sequencing results obtained after storing the ONT DNA Genomic kit at -20°C or at 4°C. The table reports the results of two independent experiments performed for each storage conditions. “Channels QC” and “Channels with Reads” indicate the number of active channels when the flow cell quality control (QC) was performed or during the sequencing, respectively. The raw read counts (Total, 2D and Pass 2D) are divided by the number of sequencing flow cell channels used during the experiment (Channels with reads) in order to normalize for the specific efficiency of each flow-cell and for the sequencing run time (Normalized Reads). Percentages are calculated by dividing the number of 2D and Pass 2D reads by the total number of reads.

#### Environmental conditions

To mimic the conditions found in a tropical forest environment, the library preparation and the sequencing were tested in the greenhouse of the Trento Science Museum, *i*.*e*. in the presence of a mean temperature of 27°C and 98% humidity. Even if the absolute number of total reads generated in the greenhouse was lower than in the laboratory test, possibly due to a lower number of active pores, the percentage of total 2D reads and 2D-pass reads were comparable between the two conditions, thus indicating similar sequencing performances (Table 4).

**Table 4:** Impact of environmental conditions on sequencing performances.

			*Raw Reads*			*Normalized Reads*		
*Environment*	Channels QC	Channels with Reads	Total	2D	Pass 2D	Total	2D	Pass 2D
***Laboratory***	365	353	115673	20529 (17.7%)	5652 (4.9%)	328	58	16
***Greenhouse***	120	128	11594	2307 (19.9%)	784 (6.8%)	91	18	6

The table reports the sequencing results obtained from experiments performed under different environmental conditions, i.e. in standard laboratory or tropical greenhouse conditions, the latter to simulate extreme environmental conditions in the field. “Channels QC” and “Channels with Reads” indicate the number of active channels when the flow cell quality control (QC) was performed or during the sequencing, respectively. The raw read counts (Total, 2D and Pass 2D) are divided by the number of sequencing flow cell channels used during the experiment (Channels with reads) in order to normalize for the specific efficiency of each flow-cell and for the sequencing run time (Normalized Reads). Percentages are calculated by dividing the number of 2D and Pass 2D reads by the total number of reads.

#### Protocols

To reduce the library preparation time as well as the usage of reagents that needed storage at -20°C, we compared the MinION sequencing results obtained using three different library preparation protocols (Table 5). The complete protocol of library preparation including the end-repair and dA-tailing steps recommended by ONT (protocol 1), was initially compared to protocol 2 that omits these steps. As expected, in the absence of phosphorylated amplicon ends, the sequencing library was not effectively generated with protocol 2, thus producing only 1/100 reads as compared to protocol 1 and only a negligible percentage of 2D sequences. Therefore, protocol 3 was further modified using 5’-end phosphorylated primers in the initial barcoding amplification step. This adjustment allowed to bypass the end-repair and dA-tailing steps but to maintain the performance of the standard protocol: the normalized number of 2D reads per channel and the 2D-pass rate were comparable between protocol 1 and 3.

**Table 5:** MinION sequencing data from experiments involving different sample preparation protocols.

				Raw Reads			Normalized Reads		
Protocol	Adjustments	Channels QC	Channels with reads	Total	2D	Pass 2D	Total	2D	Pass 2D
**1**	none	480	257	17,0193	10,250 (6.0%)	3,730 (2.2%)	662	40	15
**2**	end-repair and dA-tailing removed	344	320	1,839	5 (0.2%)	2 (0.1%)	4	0	0
**3**	end-repair and dA-tailing removed, PCR with phosphorilated primers	208	203	54,512	9,536 (17.5%)	3,441 (6.3%)	269	47	17

Protocol 1 includes dA-tailing and end-repair steps whereas Protocol 2 and 3 omit these steps; in protocol 3 PCR uses phosphorylated primers. “Channels QC” and “Channels with Reads” indicate the number of active channels when the flow cell quality control (QC) was performed or during the sequencing, respectively. Read counts (Total, 2D and Pass 2D) are divided by the number of sequencing flow cell channels used during the experiment (Channels with reads) in order to normalize for the specific efficiency of each flow-cell and for the sequencing run time (Normalized Reads). Percentages are calculated by dividing 2D and Pass 2D by the total number of reads.

#### Accuracy of the MinION

**Fig 2:**
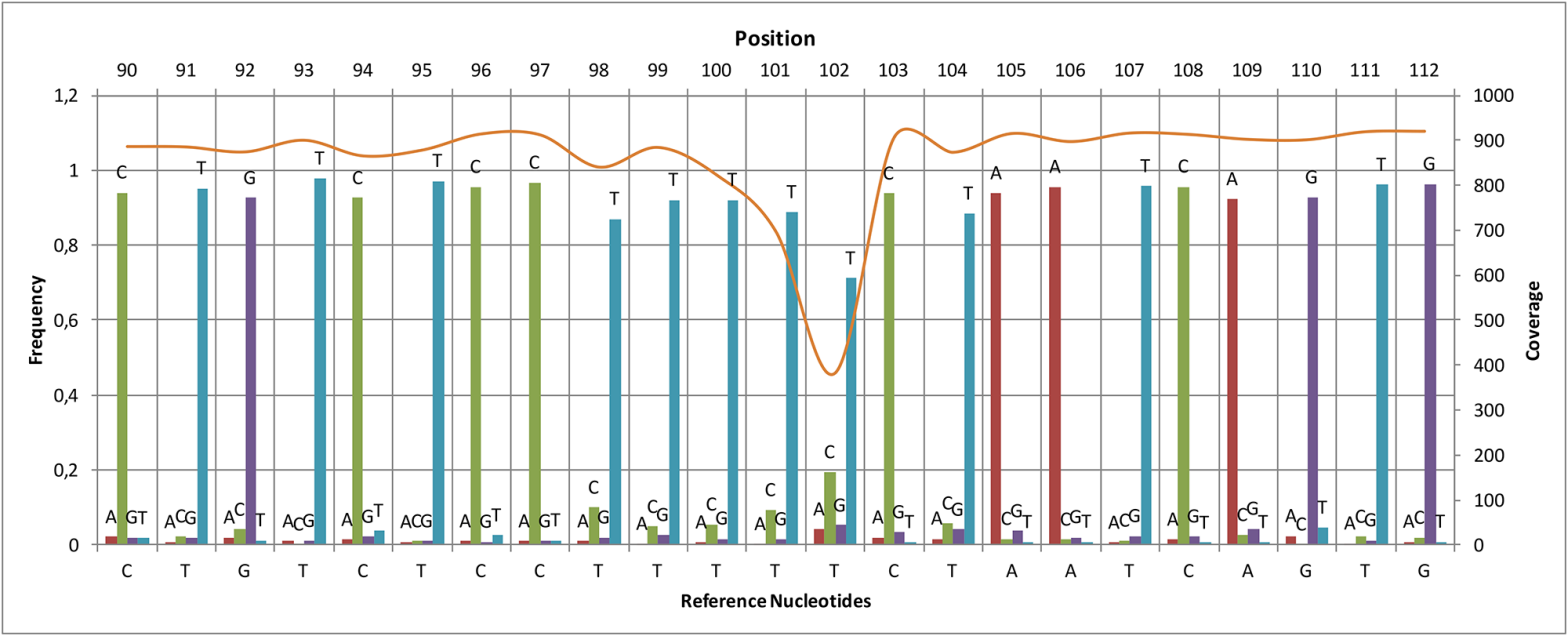
Nucleotide frequencies and coverage of aligned reads. The nucleotide frequencies (bars) of the aligned reads were calculated at every position using the Sanger sequence as reference. The minimum value of the ‘correct nucleotide’ frequency, *i*.*e*. corresponding to the reference, along the entire sequence was 0.66. The sequence coverage (continuous line) was obtained by counting the number of nucleotides aligned over each reference position. The frequency value of the four nucleotides and the coverage are shown in a region with average complexity and a homopolymer run, the latter showing a clear drop in coverage.

**Fig 3:**
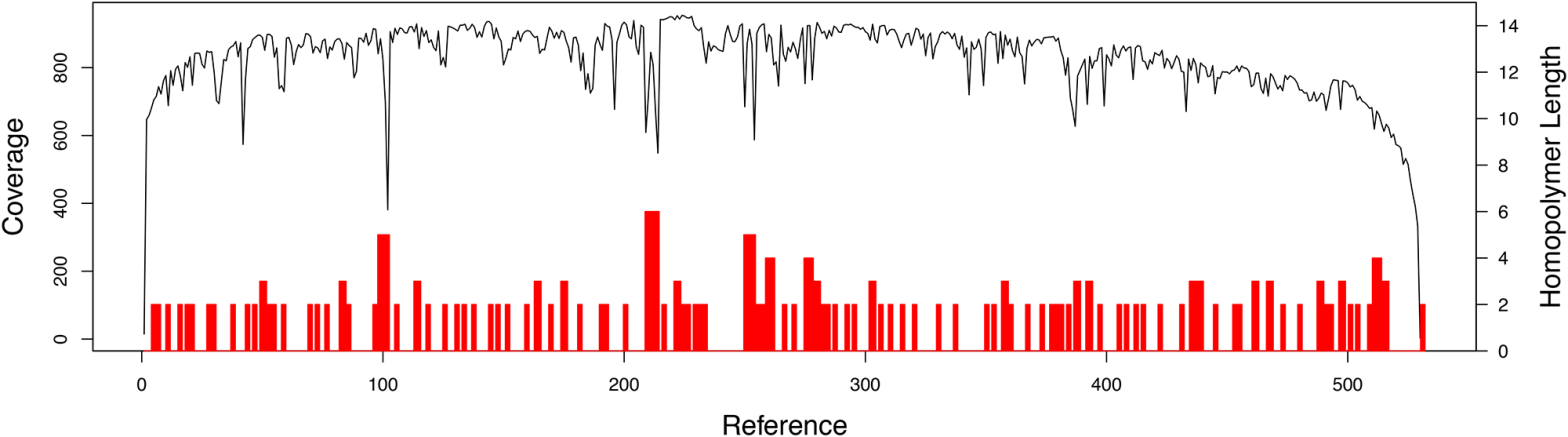
Nanopore sequencing coverage *vs* homopolymer runs. Read coverage (upper line) and homopolymer runs (bars) along the 532-bp *Amietophrynus brauni* barcode region is shown. The coverage value of MinION reads was calculated as respect to the reference sequence generated by Sanger. The homopolymer runs indicated with a bar correspond to sequences of at least two identical nucleotides.

In order to test the performance of the MinION sequencing platform, 16S amplicons of the toad, *Amietophrynus brauni*, were sequenced using the Sanger and the MinION methods in parallel. The MinION run produced 51,273 reads including 8,555 in which the template and complement were merged to obtain more accurate data (2D reads). From this dataset, 2,660 2D reads passed the quality filter set by the Metrichor Agent (2D pass) (Table 6) and 977 of these reads (37% of the 2D pass dataset) were of sufficient quality to be successfully aligned to the Sanger reference sequence. The mean error of the MinION reads was 17% when aligned to the Sanger sequence, including 8% mismatch, 4% insertion and 5% deletion. Despite these errors, we observed that the consensus sequence generated by calling the most frequent nucleotide at each position was 100% accurate when compared to the sequence generated by Sanger (Fig 2). Furthermore, in agreement with previous literature [33], the major low-coverage regions were homopolymer runs, indicating that most MinION errors consist of homopolymer-length sequencing errors. This was further confirmed by analyzing the coverage distribution along the sequence, which showed spikes of low coverage in correspondence of homopolymer stretches (Fig 3).

**Table 6:** MinION sequencing data and sequence identification results.

Sample species	Gene	Total Reads	2D Reads	2D Pass Reads	Similarity %			Reference (Accession number)
					Loman’s	ONtoBAR	Sanger	
***Amietophrynus brauni***	16S	51,273	8,555	2,660	*99%*	*100%*	*100%*	*Bufo brauni (AF220886)*
***Leptopelis vermiculatus***	16S	109,047	57,110	42,102	*92%*	*98%*	*98%*	*Leptopelis sp*. *(A168408)*
***Leptopelis vermiculatus***	CO1	181,123	113,663	110,921	*86%*	*83%*	*82%*	*S*. *araneus (JF499348)*
***Rieppeleon brachyurus***	16S	97,080	16,760	8,026	*92%*	*100%*	*100%*	*R*. *brachyurus* (voucher AG19033)
***Sorex alpinus***	16S	84,913	24,807	7,706	*98%*	99%	*99%*	*S*. *alpinus (DQ630322)*
***Rhynchocyon udzungwensis***	CO1	167,466	104,419	97,725	88%	99%	97%	*R*. *petersi* (AG19033)
***Arthroleptis xenodactyloides***	16S	5,039	187	2	97%	100%	100%	*A*. *xenodactyloides* (A137057)

For each experiment the table reports the sample species, the name of the sequenced gene, the total number of reads obtained by MinION sequencing, the number of 2D reads, and the PASS subsets. The similarity % columns show the identity scores between Loman’s consensus, ONtoBAR consensus and Sanger compared to the reference sequence reported in the last column.

Such systematic errors in the sequencing data can strongly impact the accurate determination of barcoding sequences, thus providing misleading results in the precise identification of species. To bypass this issue, we developed a more robust pipeline to obtain a reliable consensus sequence, in which the *de novo* assembly of the barcode gene is BLASTed against the NCBI nucleotide (nt) database, the most similar sequence is retrieved and used as a reference sequence to reconstruct a final consensus from the initial set of raw MinION reads that is then BLASTed again against NCBI nt database (“ONtoBAR” pipeline, Fig 1). The ONtoBAR approach was tested in six experiments in which different organisms and barcoding genes were sequenced (Table 2). We examined the CO1 gene and 16S region of an amphibian (the big-eyed tree frog, *Leptopelis vermiculatus*), the 16S regions of a toad (*Amietophrynus brauni*), a squamate reptile (the beardless pygmy chameleon, *Rieppeleon brachyurus*), a mammal (the alpine shrew, *Sorex alpinus*) and the CO1 gene of another mammal, the gray-faced sengi (*Rhynchocyon udzungwensis*). A consensus sequences was initially generated based on the Loman’s method and used as a BLAST query to retrieve the best hit from the NCBI nucleotide (nt) database (Table 6). The deposited sequence was then used as a reference to align the set of 2D MinION reads to generate the ONtoBAR consensus sequence (Table 6). The sequence identity obtained with the ONtoBAR pipeline was 100% in the case of *A*. *brauni and R*. *brachyurus*. (our sequence and the one deposited in GenBank were derived from the same individuals, museum accession number MTSN5259 and MTSN5590 respectively) or included minor differences when our sequences were from different populations *e*.*g*. *L*. *vermiculatus* and *S*. *alpinus*. or different species *e*.*g*. *R*. *udzungwensis* than those retrieved from the NCBI database.

The same identity percentages were obtained when the Sanger sequence of each sample was used as query in the blast analysis (S2 File). These results indicate that barcoding using the MinION platform in combination with the ONtoBAR pipeline has the same discrimination capacity as Sanger sequencing.

### DNA barcoding in a tropical forest

**Fig 4:**
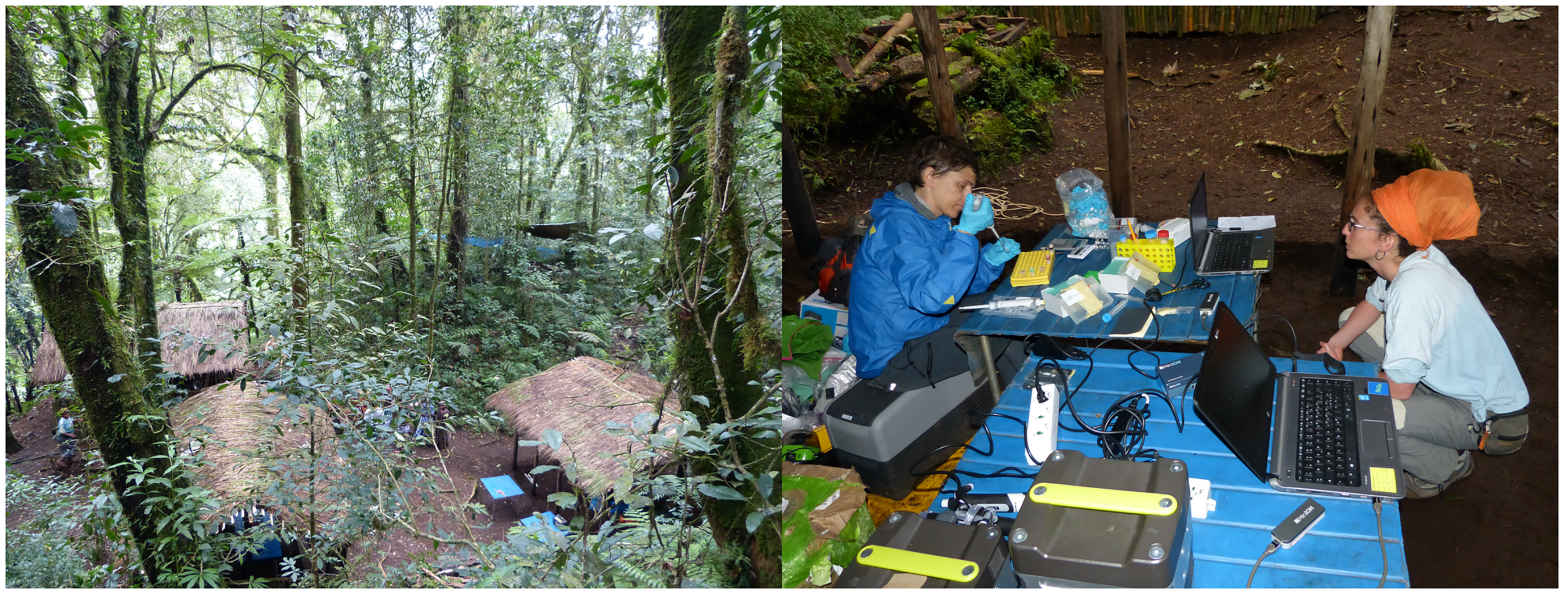
Forest field laboratory. The field laboratory set-up for conducting MinION sequencing of amphibians in a montane rainforest of Tanzania.

The portable sequencing laboratory was tested *in situ* in a montane rainforest of central-south Tanzania (mean temperature = 26°C, humidity = 98%, no electricity supply) (Fig 4).

A wild frog, belonging to the genus *Arthroleptis* according to the morphological characterization, was caught, a blood sample was taken, and the frog was subsequently released back to the nature. The *Arthroleptis’s* 16S amplification and sequencing *in situ*, within the Tanzania forest, produced a relatively low yield, with data merged from three different experiments generating 5039 reads, including 187 2D reads and only two 2D-pass reads. The Metrichor online base-calling was not performed *in situ* due to the poor signal and speed of the 2G network present in the forest, but it was properly completed after reaching a location with 3G coverage. Blasting the consensus sequence obtained by calling the most frequent nucleotide at each position *vs* the NCBI database retrieved a sequence from *Arthroleptis xenodactyloides* sharing 96% identity with the provided input (Figure A in S1 File). The PCR product generated in the field was returned to Italy and analyzed on a Sanger sequencing machine to evaluate the quality of the sequencing results generated in the rainforest. A BLAST search using the Sanger sequence confirmed that the captured frog was *A*. *xenodactyloides* (Figure B in S1 File), however, the barcode sequence produced in Tanzania by MinION had 4% mismatch when compared to the Sanger data (Figure C in S1 File).

**Fig 5:**
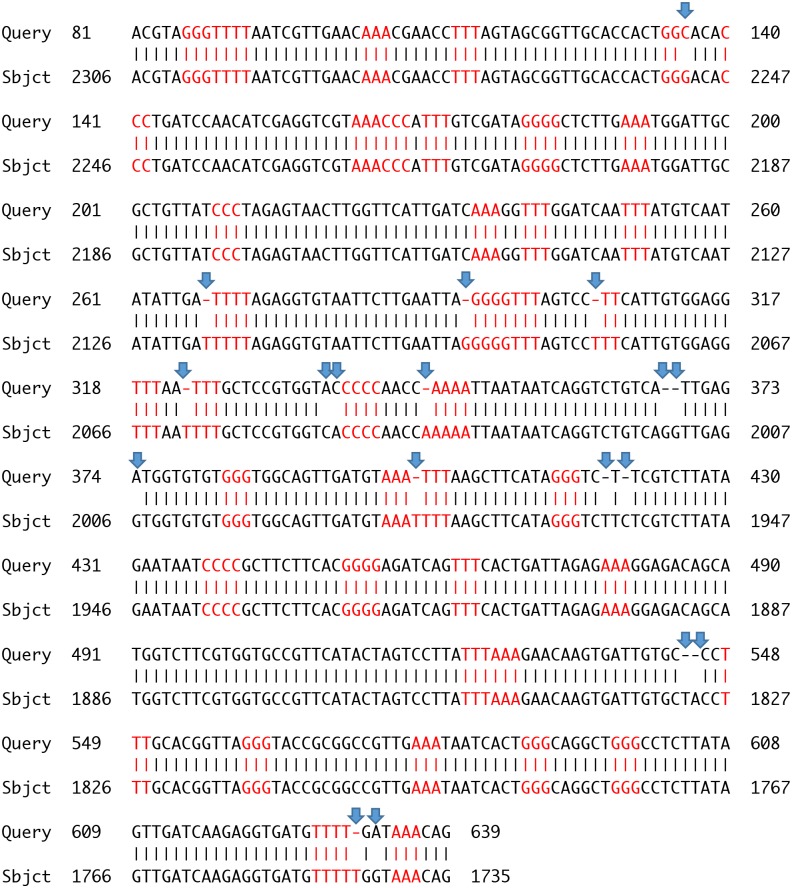
NCBI BLAST search of the assembled sequence of *Arthroleptis xenodactyloides*. Alignment result of the assembled consensus sequence of *A*. *xenodactyloides* (Query, 741 bp) with with the highest score hit retrieved from NCBI BLAST, *i*.*e*. *A*. *xenodactyloides* sequence (FJ151103.1; 2313 bp), Sbjct. The two sequences matched with 97% identity. Homopolymer sites with more than two identical consecutive nucleotides are highlighted in red while mis-matches are marked by an arrow.

The “ONtoBAR” pipeline was applied on the *A*. *xenodactyloides* MinION data generated in the field as described above, but using the whole set of 2D reads, given the low yield of 2D pass. The generated consensus sequence returned a best hit with 97% identity to the *A*. *xenodactyloides* 16*S* sequence from the NCBI nt database (Table 6). The majority of errors were found in homopolymer runs (Fig 5). The *A*. *xenodactyloides* 16*S* sequence retrieved from the NCBI nt database was then used as a reference to align the set of 187 2D MinION reads. Despite lower quality reads were used, the consensus sequence reconstructed using the ONtoBAR procedure was 100% identical to the Sanger product (Figure D in S1 File), confirming that the use of a reference similar to the species under investigation overcomes the difficulty of reconstructing the entire length of the homopolymer regions.

### Assessment of new sequencing chemistry

In order to verify the potential future development of our portable sequencing kit, we have tested the new version of the MinION sequencer (v. Mk 1B) along with the new flowcell and sequencing kit (based on chemistry R9.4) as soon as they were released. At this aim, amplicons of the 16S gene from *A*. *brauni* were used as starting material. The new MinION device and chemistry generated about 5 times more reads in the same sequencing time (about 16h); of these more than 60% had enough quality to pass the Metrichor quality filter (Table 7). Most importantly the mean error of the sequences generated was about half as compared to a previous experiment and corresponded to a lower amount of mismatches and insertions (Table 7). These results demonstrate that the portable sequencing kit has the potential to develop further following the improvement of the MinION sequencing chemistry, and thus provide even more accurate barcoding data for species identification.

**Table 7:** Comparison of sequencing data generated with the old and new MinION flowcell and chemistries.

Flowcell	Total Reads	2D Pass	Aligned Reads	Mean Error	Mismatch	Insertion	Deletion
R7.4	36,091	1,539 (4.2%)	815	13%	5%	3%	5%
R9.4 (I)	160,321	98,252 (61%)	93,476	6.5%	1.6%	0.8%	4.1%
R9.4 (II)	300,252	205,929 (69%)	195,887	7.4%	2%	0.9%	4.5%

The table reports the total number of reads and 2D reads generated with the R7.4 flowcell and MAP005 sequencing kit or with the R9.4 flowcell and the SQK-LSK208 library preparation kit, in two different days (I-II). The number of reads that could be aligned to the reference Sanger sequence is shown, along with the percentage and types of errors detected in the MinION sequences.

## Discussion

We have developed a portable kit for on-site barcode sequencing and confirmed that sequencing is feasible under tropical forest conditions. All the instruments implemented met the needs of a mobile laboratory, being battery-powered and suitcase-sized and demonstrated comparable capabilities to their benchtop counterparts. In addition, reagents and protocols were optimized to minimize the hands-on-time and the need of low temperature storage. The setup of such portable laboratory has the potential to be implemented not only in the contest of a tropical environment but, after appropriate testing, ideally in any other condition where there is the need to sequence DNA *in situ*.

The results obtained in the tropical greenhouse were comparable with those acquired in a traditional laboratory, demonstrating both repeatability and feasibility of the sequencing procedure in tropical environmental conditions. However, the field trial yielded fewer sequences than expected and the percentage of high-quality 2D reads was much lower than that achieved in the laboratory and in the greenhouse. Although the different experimental environments may have contributed towards these differences, it is notable that the conditions in the tropical greenhouse were more extreme (higher humidity and temperature) than those in the field. Therefore, it is more likely that the reagents and flow cells were affected by the unstable shipping conditions *en route* to the experiment site and thus had lower performance than expected. In the future, these issues can be avoided by ensuring that optimal handling conditions are used for the equipment and reagents. For example, the activity of the blunt ligase needed for sample preparation using the ONT DNA Sequencing kit could be preserved by preparing a dehydrated enzyme.

Our results demonstrated that the MinION platform is suitable for the acquisition of DNA sequences from biological material *in situ*, in the context of a tropical environment. Despite the high error rate, we were able to reconstruct a 100% accurate consensus sequence that allowed us to resolve the identity of a sample even when applying non-specific amphibian reference sequences. The major regions of low coverage were homopolymer runs, reflecting homopolymer-length sequencing errors that occurred either during the alignment of MinION reads to the reference, or during the generation of a consensus sequence during the *de novo* assembly of MinION reads. These errors occur because the technology is unable to determine accurately the correct number of bases called when a single nucleotide is repeated several times [33]. As shown, the quality can be significantly improved when the ONtoBAR analysis pipeline, that takes into considerations these limits, is applied. To understand if the specimen under investigation belongs to a known species, this analysis pipeline doesn’t not require any a priori information. The ‘reference sequences’ that the software uses are sequences retrieved by the software itself from a publicly available database independently of the fact that we are studying a new or a known species. In addition, the ‘reference sequences’ do not need to represent our specimens and its function is to support the appropriate reconstruction of the actual sequence. We demonstrated here that the reads quality generated by the MinION technology in the field in combination with the ONtoBAR pipeline was sufficient to determine the taxonomic identity of an organism, as recently reported for bacteria [34].

Factors that limited the experiments on the field were the need of the 3G connection to perform the base-calling and the long sequencing run required to obtain a sufficient amount of reads. However, we expect that the most recent improvements of the technology will bypass these weakness soon. We verified that the quality of reads generated with the new flow cells and chemistry is improved as well as the yield of flow-cell, thus suggesting that future experiments in the field can be accomplished with shorter sequencing time and lower number of reads. Further developments of the technology will possibly ensure quality of base calls, thereby avoiding the need of references sequences. Finally, we foresee that a 3G connection won’t be necessary any longer as a new MinION basecaller that works locally is currently available (Albacore).

DNA barcoding has been of a big interest of the scientific community for the identification of virtually any organism that possess DNA. However, while the available sequencing platforms that are commonly used in the DNA barcoding analysis, *e*.*g*. Illumina, have a limited read-length of about 600 bp, MinION overcome this problem, producing reads longer than 200Kb (www.nanoporetech.com). Longer reads usually mean higher taxonomical resolution, as more genetic information are resolved [35]. Despite using 200Kb reads is implausible for species identification on a routine basis, the ability of MinION to read long DNA sequences will potentially allow the future assembly of entire genomes anywhere in the world, without the need of transferring wild samples to standard sequencing labs.

Our study focused on vertebrates because of the relatively good coverage of comparative sequence data available (*e*.*g*. NCBI), however for other groups (*e*.*g*. bacteria, plants and invertebrates) the lack of suitable reference data may clearly hamper appropriate taxonomic identifications. However, the accumulation of genetic data is a necessary first step in all barcoding projects and the MinION described here will minimally contribute to building up this databank. On the contrary, it will allow a cross comparison among sequenced samples from any given site and provide estimates of the numbers of taxonomic units.

The experiments described herein represented the first sequence-based identification of a species in the field and offer the prospect of real-time genomic sequencing with potentially minimal geographic, economic or infrastructural constraints. An accurate estimate of the cost of MinION sequencing is not yet available, but the potential to release sequencing technology from the current large-scale centralized infrastructure is likely to significantly reduce overall costs of DNA barcoding. We also predict that the price of the MinION device itself will follow the Carlson Curve, which describes the rapid (in some cases hyper-exponential) decline in the cost of DNA sequencing as performance and throughput increase over time [36]. This development is critical when considering the spatial mismatch between regions with high biodiversity and the distribution and availability of sequencing facilities (http://omicsmaps.com/stats). The availability of Nanopore technology in biodiversity-rich countries may therefore help to address the biodiversity knowledge gap [37], thereby contributing to the prioritization of conservation measures [38].

## Conclusions

We have demonstrated the feasibility of barcode sequencing in the field, which may constitute a step forward in biodiversity research. Genetic data are increasingly the core components of both evolutionary and ecological investigations, providing valuable insights into the relationships among phyla, but only limited data have been generated concerning diversity at lower taxonomic levels. The implementation of portable sequencing kit and devices such as those described herein may help in addressing some of the missing information on biodiversity. In addition, if the quality of data generated with portable sequencing technologies will continue to improve, they could have an impact on biodiversity assessment by fastening the pace at which genetic data can be obtained, even in hostile environmental conditions. Rapid access to genetic data can support the rapid identification of taxa in biodiversity studies as well as the quantification of habitat, species and population genetic diversity–key factors for the formulation of conservation strategies.

## Supporting information

S1 File Supplemental Figure A, B, C and D. Alignments of Arthroleptis’s 16S sequences: ONT consensus sequence vs its BLASTn best hit (A), Sanger sequence and its BLASTn best hit (B), ONT consensus sequence vs Sanger (C), Sanger vs ONT sequence after applying the ONtoBAR pipeline (D).
(PDF)

S2 File Sequences dataset. Raw MinION and Sanger reads produced in the study.
(GZ) Click here for additional data file.

S3 File Scripts repository. The folder contains scripts used for the reconstruction of a consensus sequence from MinION sequencing reads.
(GZ) Click here for additional data file.

S4 File ONtoBAR. The folder contains program files and instructions of the ONtoBAR software.
(GZ) Click here for additional data file.
