# Supplementary material for: On site DNA barcoding by nanopore sequencing
Source: PLoS One. 2017 Oct 4;12(10):e0184741. doi: 10.1371/journal.pone.0184741 (PMC5627904; doi:10.1371/journal.pone.0184741)
Supplement: S1 File — Alignments of Arthroleptis’s 16S sequences: ONT consensus sequence vs its BLASTn best hit (A), Sanger sequence and its BLASTn best hit (B), ONT consensus sequence vs Sanger (C), Sanger vs ONT sequence after applying the ONtoBAR pipeline (D). (PDF) [file pone.0184741.s001.pdf]

```
Query  1     TCGCCTGTTTATCAAAAACATCACCTCTTGATCAAACTATAAGAGGCCCAGCCTGCCCAG  60
             ||||||||||| ||||||||||||||||||||| ||||||||||||||||||||||||||
Sbjct  1731  TCGCCTGTTTACCAAAAACATCACCTCTTGATC-AACTATAAGAGGCCCAGCCTGCCCAG  1789

Query  61    TGATTATTTCAACGGCCGCGGTACCCTAACCGTGCAAAGGTAGCACAATCACTTGTTCTT  120
             ||||||||||||||||||||||||||||||||||||||||||||||||||||||||||||
Sbjct  1790  TGATTATTTCAACGGCCGCGGTACCCTAACCGTGCAAAGGTAGCACAATCACTTGTTCTT  1849

Query  121   TAAATAAGGACTAGTATGAACGGCACCACGAAGACCATGCTGTCTCCTTTCTCTAATCAG  180
             ||||||||||||||||||||||||||||||||||||||||||||||||||||||||||||
Sbjct  1850  TAAATAAGGACTAGTATGAACGGCACCACGAAGACCATGCTGTCTCCTTTCTCTAATCAG  1909

Query  181   TGAAACTGATCTCCCCGTGAAGAAGCGGGGATTATTCTATAAGACGAGAAGACCCTATGA  240
             ||||||||||||||||||||||||||||||||||||||||||||||||||||||||||||
Sbjct  1910  TGAAACTGATCTCCCCGTGAAGAAGCGGGGATTATTCTATAAGACGAGAAGACCCTATGA  1969

Query  241   AGCTTCAAATTTACATCAACTGCCACCTAAACACACCACCTCAACTCGACAGACCTGATT  300
             ||||| ||||||||||||||||||||| |  ||||||||||||||  |||||||||||||
Sbjct  1970  AGCTTAAAATTTACATCAACTGCCACCCA--CACACCACCTCAACCTGACAGACCTGATT  2027

Query  301   ATTAATTTTCGGTTGGGGTGACCACGGAGCAAA-TTAAAGCCTCCACAATGAAAGGGACT  359
             ||||||||| ||||||||||||||||||||||| ||||| |||||||||||||||| |||
Sbjct  2028  ATTAATTTTTGGTTGGGGTGACCACGGAGCAAAATTAAA-CCTCCACAATGAAAGG-ACT  2085

Query  360   AAACCCC-TAATTCAAGAATTACACCTCTAAAAATCAAAATATTGACATAAATTGATCCA  418
             ||||||| |||||||||||||||||||||||||||||| |||||||||||||||||||||
Sbjct  2086  AAACCCCCTAATTCAAGAATTACACCTCTAAAAATCAATATATTGACATAAATTGATCCA  2145

Query  419   AACCCATATTGATCAATGAACCAAGTTACTCTGGGGATAACAGCGCAATCCATTTCAACA  478
             ||||  | |||||||||||||||||||||||| ||||||||||||||||||||||||| |
Sbjct  2146  AACC--T-TTGATCAATGAACCAAGTTACTCTAGGGATAACAGCGCAATCCATTTCAAGA  2202

Query  479   GCCCCTATCGACAAATGGGTTTACGACCTCGATGTTGGATCAGGGTGTCCCAGTGGTGCA  538
             ||||||||||||||||||||||||||||||||||||||||||||||||||||||||||||
Sbjct  2203  GCCCCTATCGACAAATGGGTTTACGACCTCGATGTTGGATCAGGGTGTCCCAGTGGTGCA  2262

Query  539   ACTACTACTAAAGGTTCGTTTGTTCAACGATTAAAACCCTACGTGATCTG  588
             ||  ||||||||||||||||||||||||||||||||||||||||||||||
Sbjct  2263  ACCGCTACTAAAGGTTCGTTTGTTCAACGATTAAAACCCTACGTGATCTG  2312
```

Figure A. Alignment between Arthroleptis's 16S ONT consensus sequence and its BLASTn best hit.

Alignment of the consensus sequence obtained in the field after MinION sequencing (Query, 588 bp) with the highest score hit (96%) retrieved from NCBI BLAST (*Arthroleptis xenodactyloides*, Sbjct).

```
Query  1     ATCACCTCTTGATCAACTATAAGAGGCCCAGCCTGCCCAGTGATTATTTCAACGGCCGCG  60
             ||||||||||||||||||||||||||||||||||||||||||||||||||||||||||||
Sbjct  1750  ATCACCTCTTGATCAACTATAAGAGGCCCAGCCTGCCCAGTGATTATTTCAACGGCCGCG  1809

Query  61    GTACCCTAACCGTGCAAAGGTAGCACAATCACTTGTTCTTTAAATAAGGACTAGTATGAA  120
             ||||||||||||||||||||||||||||||||||||||||||||||||||||||||||||
Sbjct  1810  GTACCCTAACCGTGCAAAGGTAGCACAATCACTTGTTCTTTAAATAAGGACTAGTATGAA  1869

Query  121   CGGCACCACGAAGACCATGCTGTCTCCTTTCTCTAATCAGTGAAACTGATCTCCCCGTGA  180
             ||||||||||||||||||||||||||||||||||||||||||||||||||||||||||||
Sbjct  1870  CGGCACCACGAAGACCATGCTGTCTCCTTTCTCTAATCAGTGAAACTGATCTCCCCGTGA  1929

Query  181   AGAAGCGGGGATTATTCTATAAGACGAGAAGACCCTATGAAGCTTAAAATTTACATCAAC  240
             ||||||||||||||||||||||||||||||||||||||||||||||||||||||||||||
Sbjct  1930  AGAAGCGGGGATTATTCTATAAGACGAGAAGACCCTATGAAGCTTAAAATTTACATCAAC  1989

Query  241   TGCCACCCACACACCACCTCAACCTGACAGACCTGATTATTAATTTTTGGTTGGGGTGAC  300
             ||||||||||||||||||||||||||||||||||||||||||||||||||||||||||||
Sbjct  1990  TGCCACCCACACACCACCTCAACCTGACAGACCTGATTATTAATTTTTGGTTGGGGTGAC  2049

Query  301   CACGGAGCAAAATTAAACCTCCACAATGAAAGGACTAAACCCCCTAATTCAAGAATTACA  360
             ||||||||||||||||||||||||||||||||||||||||||||||||||||||||||||
Sbjct  2050  CACGGAGCAAAATTAAACCTCCACAATGAAAGGACTAAACCCCCTAATTCAAGAATTACA  2109

Query  361   CCTCTAAAAATCAATATATTGACATAAATTGATCCAAACCTTTGATCAATGAACCAAGTT  420
             ||||||||||||||||||||||||||||||||||||||||||||||||||||||||||||
Sbjct  2110  CCTCTAAAAATCAATATATTGACATAAATTGATCCAAACCTTTGATCAATGAACCAAGTT  2169

Query  421   ACTCTAGGGATAACAGCGCAATCCATTTCAAGAGCCCCTATCGACAAATGGGTTTACGAC  480
             ||||||||||||||||||||||||||||||||||||||||||||||||||||||||||||
Sbjct  2170  ACTCTAGGGATAACAGCGCAATCCATTTCAAGAGCCCCTATCGACAAATGGGTTTACGAC  2229

Query  481   CTCGATGTTGGATCAGGGTGTCCCAGTGGTGCAACCGCTACTAAAGGTTCGTTTGTTCAA  540
             ||||||||||||||||||||||||||||||||||||||||||||||||||||||||||||
Sbjct  2230  CTCGATGTTGGATCAGGGTGTCCCAGTGGTGCAACCGCTACTAAAGGTTCGTTTGTTCAA  2289

Query  541   CGATTAAAACCCTACGTGATCT  562
             ||||||||||||||||||||||
Sbjct  2290  CGATTAAAACCCTACGTGATCT  2311
```

Figure B. Alignment between Arthroleptis's 16S Sanger sequence and its BLASTn best hit. Alignment of the Sanger sequence of the sample amplified in the field (562 bp) with the highest score hit (100%) retrieved from NCBI BLAST (*Arthroleptis xenodactyloides*, Sbjct).

```
Query  20   ATCACCTCTTGATCAAACTATAAGAGGCCCAGCCTGCCCAGTGATTATTTCAACGGCCGC  79
            |||||||||||||| |||||||||||||||||||||||||||||||||||||||||||||
Sbjct  1    ATCACCTCTTGATC-AACTATAAGAGGCCCAGCCTGCCCAGTGATTATTTCAACGGCCGC  59

Query  80   GGTACCCTAACCGTGCAAAGGTAGCACAATCACTTGTTCTTTAAATAAGGACTAGTATGA  139
            ||||||||||||||||||||||||||||||||||||||||||||||||||||||||||||
Sbjct  60   GGTACCCTAACCGTGCAAAGGTAGCACAATCACTTGTTCTTTAAATAAGGACTAGTATGA  119

Query  140  ACGGCACCACGAAGACCATGCTGTCTCCTTTCTCTAATCAGTGAAACTGATCTCCCCGTG  199
            ||||||||||||||||||||||||||||||||||||||||||||||||||||||||||||
Sbjct  120  ACGGCACCACGAAGACCATGCTGTCTCCTTTCTCTAATCAGTGAAACTGATCTCCCCGTG  179

Query  200  AAGAAGCGGGGATTATTCTATAAGACGAGAAGACCCTATGAAGCTTCAAATTTACATCAA  259
            |||||||||||||||||||||||||||||||||||||||||||||| |||||||||||||
Sbjct  180  AAGAAGCGGGGATTATTCTATAAGACGAGAAGACCCTATGAAGCTTAAAATTTACATCAA  239

Query  260  CTGCCACCTAAACACACCACCTCAACTCGACAGACCTGATTATTAATTTTCGGTTGGGGT  319
            |||||||| |  ||||||||||||||  |||||||||||||||||||||| |||||||||
Sbjct  240  CTGCCACCCA--CACACCACCTCAACCTGACAGACCTGATTATTAATTTTTGGTTGGGGT  297

Query  320  GACCACGGAGCAAA-TTAAAGCCTCCACAATGAAAGGGACTAAACCCC-TAATTCAAGAA  377
            |||||||||||||| ||||| |||||||||||||||| |||||||||| |||||||||||
Sbjct  298  GACCACGGAGCAAAATTAAA-CCTCCACAATGAAAGG-ACTAAACCCCCTAATTCAAGAA  355

Query  378  TTACACCTCTAAAAATCAAAATATTGACATAAATTGATCCAAACCCATATTGATCAATGA  437
            ||||||||||||||||||| |||||||||||||||||||||||||  | |||||||||||
Sbjct  356  TTACACCTCTAAAAATCAATATATTGACATAAATTGATCCAAACC--T-TTGATCAATGA  412

Query  438  ACCAAGTTACTCTGGGGATAACAGCGCAATCCATTTCAACAGCCCCTATCGACAAATGGG  497
            ||||||||||||| ||||||||||||||||||||||||| ||||||||||||||||||||
Sbjct  413  ACCAAGTTACTCTAGGGATAACAGCGCAATCCATTTCAAGAGCCCCTATCGACAAATGGG  472

Query  498  TTTACGACCTCGATGTTGGATCAGGGTGTCCCAGTGGTGCAACTACTACTAAAGGTTCGT  557
            |||||||||||||||||||||||||||||||||||||||||||  |||||||||||||||
Sbjct  473  TTTACGACCTCGATGTTGGATCAGGGTGTCCCAGTGGTGCAACCGCTACTAAAGGTTCGT  532

Query  558  TTGTTCAACGATTAAAACCCTACGTGATCT  587
            ||||||||||||||||||||||||||||||
Sbjct  533  TTGTTCAACGATTAAAACCCTACGTGATCT  562
```

Figure C. Alignment between the sequences of Arthroleptis's 16S generated by ONT and Sanger. Alignment of the consensus sequence obtained in the field after MinION sequencing (Query, 587 bp) with Sanger sequence (Sbjct, 562 bp), showing 96% identity score.

```
Query  1    ATCACCTCTTGATCAACTATAAGAGGCCCAGCCTGCCCAGTGATTATTTCAACGGCCGCG  60
            ||||||||||||||||||||||||||||||||||||||||||||||||||||||||||||
Sbjct  50   ATCACCTCTTGATCAACTATAAGAGGCCCAGCCTGCCCAGTGATTATTTCAACGGCCGCG  109

Query  61   GTACCCTAACCGTGCAAAGGTAGCACAATCACTTGTTCTTTAAATAAGGACTAGTATGAA  120
            ||||||||||||||||||||||||||||||||||||||||||||||||||||||||||||
Sbjct  110  GTACCCTAACCGTGCAAAGGTAGCACAATCACTTGTTCTTTAAATAAGGACTAGTATGAA  169

Query  121  CGGCACCACGAAGACCATGCTGTCTCCTTTCTCTAATCAGTGAAACTGATCTCCCCGTGA  180
            ||||||||||||||||||||||||||||||||||||||||||||||||||||||||||||
Sbjct  170  CGGCACCACGAAGACCATGCTGTCTCCTTTCTCTAATCAGTGAAACTGATCTCCCCGTGA  229

Query  181  AGAAGCGGGGATTATTCTATAAGACGAGAAGACCCTATGAAGCTTAAAATTTACATCAAC  240
            ||||||||||||||||||||||||||||||||||||||||||||||||||||||||||||
Sbjct  230  AGAAGCGGGGATTATTCTATAAGACGAGAAGACCCTATGAAGCTTAAAATTTACATCAAC  289

Query  241  TGCCACCCACACACCACCTCAACCTGACAGACCTGATTATTAATTTTTGGTTGGGGTGAC  300
            ||||||||||||||||||||||||||||||||||||||||||||||||||||||||||||
Sbjct  290  TGCCACCCACACACCACCTCAACCTGACAGACCTGATTATTAATTTTTGGTTGGGGTGAC  349

Query  301  CACGGAGCAAAATTAAACCTCCACAATGAAAGGACTAAACCCCCTAATTCAAGAATTACA  360
            ||||||||||||||||||||||||||||||||||||||||||||||||||||||||||||
Sbjct  350  CACGGAGCAAAATTAAACCTCCACAATGAAAGGACTAAACCCCCTAATTCAAGAATTACA  409

Query  361  CCTCTAAAAATCAATATATTGACATAAATTGATCCAAACCTTTGATCAATGAACCAAGTT  420
            ||||||||||||||||||||||||||||||||||||||||||||||||||||||||||||
Sbjct  410  CCTCTAAAAATCAATATATTGACATAAATTGATCCAAACCTTTGATCAATGAACCAAGTT  469

Query  421  ACTCTAGGGATAACAGCGCAATCCATTTCAAGAGCCCCTATCGACAAATGGGTTTACGAC  480
            ||||||||||||||||||||||||||||||||||||||||||||||||||||||||||||
Sbjct  470  ACTCTAGGGATAACAGCGCAATCCATTTCAAGAGCCCCTATCGACAAATGGGTTTACGAC  529

Query  481  CTCGATGTTGGATCAGGGTGTCCCAGTGGTGCAACCGCTACTAAAGGTTCGTTTGTTCAA  540
            ||||||||||||||||||||||||||||||||||||||||||||||||||||||||||||
Sbjct  530  CTCGATGTTGGATCAGGGTGTCCCAGTGGTGCAACCGCTACTAAAGGTTCGTTTGTTCAA  589

Query  541  CGATTAAAACCCTACGTGATCT  562
            ||||||||||||||||||||||
Sbjct  590  CGATTAAAACCCTACGTGATCT  611
```

Figure D. Alignment between the sequences of Arthroleptis's 16S as generated by Sanger and by ONT after applying the ONtoBAR pipeline.

Alignment of Sanger sequence (Query, 562 bp) with the consensus sequence (Sbjct, 611 bp) obtained after applying the ONtoBAR pipeline on the MinION data generated in the field, showing 100% identity score.
