# Supplementary material for: On site DNA barcoding by nanopore sequencing
Source: PLoS One. 2017 Oct 4;12(10):e0184741. doi: 10.1371/journal.pone.0184741 (PMC5627904; doi:10.1371/journal.pone.0184741)
Supplement: S4 File — The folder contains program files and instructions of the ONtoBAR software. (GZ) [file pone.0184741.s004.tar.gz › ONtoBAR/README.html]

# ONtoBAR

ONtoBAR, a two-step pipeline for MinION-based DNA barcoding
that

1. retrieves from the NCBI nt database the reference sequence that
   is most similar to the consensus sequence obtained by the de novo
   assembly of MinION reads
2. calls variants by aligning the MinION reads against the
   reference sequence.

## INSTALL

De-compress the **ONtoBAR** archive and install all
the required softwares:

- the blastN software and a local copy of the NT database
- poretools from http://poretools.readthedocs.org/en/latest/
- nanocorrect from https://github.com/jts/nanocorrect/
- samtools from http://www.htslib.org/
- the Whole-Genome Shotgun Assembler from
  http://sourceforge.net/projects/wgs-assembler/files/wgs-assembler/wgs-8.1/
- the python scipy library
- the LAST aligner from http://last.cbrc.jp/

## Run ONtoBAR

To run the software the user must prepare a directory for the
project (ie. *test*) and a sub-directory *FAST5*
containing the 2D PASS raw reads in FAST5 format. The program works
also if the fasta file
*/.../test/assembly/raw.reads.unsorted* is available.

To run correctly the script thw user have to pass the absolute
path of the **ONtoBAR.pl** program and the absolute
path ph the *test* diectory.
